# Miasmatic Paralytic Fever

**Published:** 1895-07

**Authors:** 


"MIASMATIC PARALYTIC FEVER."—Apropos of Dr. Peeples' paper, we would like to have the opinion and experience of our readers on the subject; would like to have it discussed through the JOURNAL, and an invitation to do so is extended to our readers.

The picture is strongly drawn; and from the doctor's experience and description, the fever must be something terrible. It seems to demand at least very prompt treatment, whether "heroic'

or not, is a matter of opinion, in which leading practitioners differ;—if we are to judge by the papers of Dr. W. W. Walker, Dr. T. F. Oates and others, on Hæmatinuria, which is possibly the same disease described by Dr. P., modified by local conditions. Personally the writer has had no experience with this fever; or if so, does not recognize it in the description given by Dr. Peeples. In the malarial sections of Mississippi and Louisiana, a disease very closely resembling that described by Dr. Peeples, occurs, and in some sections is not unfrequently met with. It is generally known there as "congestive chill," or pernicious fever, and is very fatal; but unlike the one described, is more fatal with negroes than with whites. Should the patient survive the onset, or "chill" stage, and reaction set up in due time, it is apt to be followed by a long siege of a low form of fever, closely resembling the typhoid fever as it occurred in the hospitals during the war,—which the writer can testify, was in many respects different from the typhoid fever of to-day. On the Mississippi cotton plantations, quinine was as staple a household commodity as sugar and coffee; and was the dependence, with calomel, of course, in "congestive" chill. The "overseer" dispensed it, usually.

Again, all experienced physicians in Texas are familiar with a form of fever which differs materially, in its mode of onset, from that described by Dr. Peeples, beginning usually as light remittent, which, however, does not yield to quinine, and in which occurs, after a week or ten days, or less, a seeming paralysis of the intestines; i. e., there will be, perhaps, frequent attempts at stool, but no fecal matter or mucus is passed, but only a thin serous fluid, and the torpid state of the bowels is attended with tympanitis. This fever is variously called "typho-malarial," "continued fever," slow fever, and by many, typhoid fever; Beall and West, we believe, claiming that all continued fever in Texas is "typhoid." The terms continued fever and slow fever indicate nothing and should not be used, but they constitute the most common and most popular appellation in the country districts.

Surely Dr. Peeples' experience is not unique,—surely he is not the only physician who has seen this fever; but is a little remarkable that, so far as we know, he is the first one to describe such a disease. Malarial hæmaturia, or hæmatinuria, has been written up frequently, and the doctors have quarrelled over the treatment; but from accounts it has nowhere been as fatal as the form of fever which Dr. Peeples says occurs with such frequency in Grimes county. We will be glad to hear from practitioners whose lot has thrown them in the miasmatic sections of Texas. It is to be hoped this disease is not of frequent occurrence; we have not heard of it, as described by the doctor, elsewhere than at Navasota.

